# A Review of Smoking Cessation Interventions: Efficacy, Strategies for Implementation, and Future Directions

**DOI:** 10.7759/cureus.52102

**Published:** 2024-01-11

**Authors:** Chidera N Onwuzo, John Olukorode, Walid Sange, Dolapo A Orimoloye, Chidinma Udojike, Lisa Omoragbon, Abdulraheem E Hassan, David M Falade, Raymond Omiko, Oluwatobi S Odunaike, Paul A Adams-Momoh, Ehizobhen Addeh, Somtochukwu Onwuzo, Urim Joseph-Erameh

**Affiliations:** 1 Internal Medicine, Benjamin S. Carson College of Health and Medical Sciences, Ilishan-Remo, NGA; 2 Internal Medicine, General Hospital Lagos Island, Lagos, NGA; 3 Internal Medicine, K. J. Somaiya Medical College and Hospital, Mumbai, IND; 4 Internal Medicine, College of Medicine University of Lagos, Lagos, NGA; 5 Internal Medicine, Cleveland Clinic Foundation, Cleveland, USA

**Keywords:** socioeconomic factors, pharmacogenetics, cultural diversity, tobacco epidemic, smoking cessation, smoking

## Abstract

In an era marked by increasing awareness of the detrimental effects of smoking on our health, the efficacy of smoking cessation strategies is of great significance. Numerous studies have demonstrated the effectiveness and success rates of various pharmacological and behavioral interventions, and different strategies have been proposed to optimize successful implementation. As we battle the global tobacco epidemic, it is important to better understand how to support individuals looking toward a smoke-free life.

This review commences by highlighting the burden of smoking as a public health concern, exploring various smoking cessation interventions, and assessing their effectiveness and success rates. Our attention then shifts toward strategies for putting these interventions into action while highlighting challenges in implementation, ranging from individual to socioeconomic factors. Furthermore, this review sheds light on the need to tailor interventions to suit diverse populations, taking varying individual characteristics into account. We conclude this review by discussing future directions and emerging trends, considering the roles modern technology and policies can play in aiding smoking cessation.

## Introduction and background

The global tobacco epidemic is one of the most significant public health challenges, claiming the lives of over 8 million people annually. Of these fatalities, more than 7 million stem from direct tobacco consumption, with an additional 1.3 million attributed to non-smokers exposed to second-hand smoke [1].

Smoking remains one of the most pressing global public health concerns. It is a significant risk factor for a wide range of diseases, affecting both smokers and second-hand smokers. Its effects extend beyond the respiratory system, affecting other vital organs. The link between smoking and cardiovascular diseases and cancers is well-established. Cigarette smoking is the number one risk factor for lung cancer and is linked to 80-90% of lung cancer deaths [2].

As society continues to battle tobacco addiction, the efficacy of various methods of smoking cessation becomes a vital area of research. Various strategies, including behavioral and pharmacological, as well as a combination of both [3], have shown different levels of efficacy in smoking cessation. This review aims to comprehensively assess the effectiveness of these strategies and explore ways to facilitate their successful implementation.

## Review

Types of smoking cessation interventions

In a landscape where awareness of the perils of smoking is steadily rising, an increasing number of individuals are making earnest attempts to quit this harmful habit. The primary approach to smoking cessation continues to be pharmacological interventions, behavioral therapy, and a combination of both. We briefly expand on these various methods below.

Pharmacological Therapy

Pharmacotherapy plays a pivotal role in supporting individuals on their journey to quit smoking, offering a diverse array of pharmaceutical options designed to alleviate nicotine withdrawal symptoms, curb cravings, and enhance the prospects of successful smoking cessation. Within this domain, nicotine replacement therapy (NRT), bupropion, and varenicline emerge as key players.

The U.S. Food and Drug Administration (FDA) has approved five distinct NRT products, encompassing patches, lozenges, gum, inhalers, and nasal sprays, alongside bupropion and varenicline, as integral components of smoking cessation interventions [4]. Recent randomized controlled trials have underscored the efficacy of NRT, demonstrating its superiority over a placebo [5]. Bupropion and varenicline, both targeting nicotine receptors in the brain, contribute to effective withdrawal symptom management. For a comprehensive approach to quitting, these medications are frequently complemented by counseling or participation in support groups. Nevertheless, due to potential side effects and interactions with other medications, consultation with a healthcare provider remains indispensable. It is essential to acknowledge that pharmacotherapy substantially elevates the likelihood of achieving successful smoking cessation, representing a cornerstone in the pursuit of a smoke-free life.

Behavioral Therapy

Behavioral therapy is a non-pharmacological method that employs cognitive behavioral therapy techniques, motivational interviewing, and incentives to motivate and reinforce behavior change. These strategies are often used in combination and are based on principles of behavioral and cognitive psychology. They aim to enhance smokers’ self-control over their smoking behavior by assisting them in changing unhelpful cognitions (i.e., thoughts, beliefs, and attitudes) and by structuring efforts to alter smoking behavior through various techniques, such as goal setting (e.g., establishing a quit day), self-monitoring (e.g., keeping records to identify times and places where someone smokes), providing rewards, and offering skill training to help smokers learn, practice, and implement techniques that enable them to resist urges to smoke after quitting [4].

Combination Therapy

Combination therapy utilizes both the behavioral and pharmacological approach, involving brief interventions delivered by a doctor or nurse, consisting of advice to stop smoking, combined with pharmacotherapy. It is typically the most common method through which smokers seek assistance to quit smoking [4]. A recurring challenge faced by physicians is the often limited motivation of patients to quit smoking, despite more than 60% expressing a desire to do so. However, incorporating psychotherapeutic strategies designed to manage negative effects can enhance smoking cessation interventions, boost treatment retention, and improve overall outcomes [6].

Efficacy of smoking cessation interventions

Different smoking cessation interventions have emerged with varying efficacy and success rates. Smoking cessation interventions can be either pharmacological or behavioral and can involve single therapy use or a combination of various therapies. The following scientific evidence substantiates these assertions.

Success Rates of Different Interventions

In their comprehensive study on smoking cessation interventions, Laniado-Laborín illuminated a notable disparity in success rates. They found that employing a combination of pharmacological methods and behavioral support, referred to as combination therapy, resulted in a significantly higher success rate of approximately 24% within a one-year timeframe. In contrast, relying solely on behavioral intervention yielded a comparatively lower success rate at 7%-16%. Moreover, attempting to quit smoking without any structured approach yielded a mere 3%-5% success rate within the same timeframe [7]. This study highlights the efficacy of combination therapy as a potent strategy for enhancing smoking cessation outcomes.

Giulietti et al., in their research, shed light on the enhanced efficacy of combining NRT strategies. One study within their review disclosed that augmenting a nicotine patch with fast-acting NRT resulted in a 5% increase in abstinence rates when compared to the use of NRT as a monotherapy [8]. Furthermore, their investigation unveiled that varenicline, a partial agonist of nicotinic receptors in the central nervous system known for its role in reducing cravings and aiding smoking cessation, exhibited greater effectiveness when employed in conjunction with either behavioral therapy or NRT, as opposed to its use in isolation [8]. These findings highlight the advantages of combination approaches in optimizing smoking cessation outcomes.

Gonzales et al. showed that varenicline was more effective than bupropion and placebo in smoking cessation over a four-week period with an abstinence rate of 44% with varenicline compared to 29.5% and 17.7% abstinence rate among patients on bupropion and placebo, respectively [9]. A meta-analysis conducted by Mishra et al. (2021) included 97 studies. They found that a nicotine receptor agonist (varenicline) is more effective than bupropion and NRT when used as monotherapy in smoking cessation. Moreover, the combination of varenicline and NRT produced the most effective results with an odds ratio of 4.4 (95% CI = 2.2-8.7) [10].

Overall, these studies indicated that combining two or more therapies, whether pharmacological or behavioral, yielded greater success rates compared to individual therapies [7,8]. Furthermore, the combination of a nicotine receptor agonist and replacement therapy is the most effective [9,10].

Factors Influencing Effectiveness of Smoking Cessation Interventions

The implementation of various smoking cessation methods is influenced by a multitude of factors, encompassing environmental, pharmacogenetic, individual, psychosocial, socioeconomic, and more.

Perez-Pramo and Lazarus unveiled a significant connection between an individual’s ability to quit smoking and their response to smoking cessation pharmacotherapy, influenced by hereditary variations in metabolic and nicotine receptor genes. Additionally, it was discovered that genetic factors account for approximately 50% of the variability in smoking cessation success. For instance, patients with variant alleles of the dopamine D2 receptor tend to exhibit a higher quit rate when using bupropion compared to those without these variants [11].

Individual factors play a significant role in smoking cessation. In a study using structural equation modeling, Businelle et al. found that poor social support, living in disadvantaged neighborhoods, and ineffective stress-coping mechanisms are key factors hindering smoking cessation [12]. Although pharmacological interventions are commonly employed to aid in quitting smoking, several major barriers, as identified by Guirguis et al., contribute to discontinuation, including the challenge of breaking the habit despite treatment, concerns about excessive weight gain, stress, and the potential side effects of medications [13].

Strategies for implementation

Having already discussed the effectiveness of smoking cessation interventions and understanding the factors influencing their success, our attention now turns to strategies for implementing these interventions. The discussion will be divided into two main categories: hospital-based interventions and community-based interventions.

Hospital-Based Interventions

Hospital admissions offer a unique chance for physicians to promote smoking cessation. While brief advice during this time is highly effective, it must be integrated with comprehensive cessation treatments for long-term success [14]. The primary approach involves staff education, engaging various healthcare professionals, including physicians and burses, as well as volunteers and other frontline staff to ensure proficiency.

Additionally, the adoption of technology can play a crucial role in sustaining smoking cessation interventions for the medium-to-long term. This includes incorporating prompts into patient charts for known smokers, utilizing Electronic Medical Records, and sending notifications to healthcare providers [14]. Furthermore, increasing the number of specialized staff dedicated to providing smoking cessation solutions or allocating more working hours for hospital staff to focus on these solutions is essential. Adequate financial resources are pivotal for the successful implementation of hospital-based smoking cessation interventions. Although government funding is the norm, there have been instances of internally raised resources for system and structure development [14]. It is imperative to invest in multi-strategic approaches to achieve long-term success in reducing smoking prevalence.

Community-Based Interventions

Furthermore, community-based Interventions represent opportunities to involve behavioral means of inhibiting these habits. One of such interventions is the Courage-To-Quit (CTQ) program implemented in a cohort study that measured the feasibility and effectiveness of such in a racially diverse urban smoking community. It is a semi-structured intervention comprising both orientation and psycho-education sessions with a predetermined duration dependent on the variant employed [15]. Feasibility rates (75%) and acceptability rates (95%) underline its pragmatic nature while outcomes for the likelihood of quitting smoking at at 36%, which is consistent with tobacco treatment guidelines [15]. Along with excellent economic implications, the above suggests the CTQ Program is an excellent community-based behavioral intervention. Others include personal smoking cessation therapy, quit classes, and telephone quit lines [15].

Through a combination of hospital- and community-based programs, it is feasible to create multi-strategic solutions that would aid in the reduction of smoking prevalence, improve smoking cessation rates, and create sustainable working plans for long-term success.

Challenges in implementation

The multifaceted efforts applied globally aimed at reducing smoking and achieving eventual cessation have yielded significant results. However, a gap between policies, implementation, and the expected results exists, prompting a review of the challenges and the need for appropriate actions.

Healthcare personnel, who are situated in the midst of implementation, have expressed various challenges during their course of work. Increased patient load and burden, along with short patient encounter times, reduce opportunities for meaningful interactions and adequate time for exploring patient concerns and providing counseling on various methods available to encourage cessation [16].

Inconsistencies in reiterating the protocols and guidelines for the healthcare personnel have also created some laxity in the implementation as the goal and smoking burden are not emphasized enough and clinician knowledge is not refreshed [16].

On the other hand, patients have been shown to experience a lack of proper incentives to stop smoking, including follow-up care, affordable smoking alternatives, adequate information on available smoking cessation options, and empathy from clinicians. Another limitation that patients experience is insufficient participation in group-based cessation interventions, especially among their peers. Due to patient-only focused interventions, when individuals return to their communities without achieving smoking cessation, they can be influenced by peer pressure and return to smoking [17].

Disparities in the implementation of smoking cessation programs are influenced by socioeconomic factors such as income status, gender, and associated comorbidities. Research conducted by O'Connell et al. has revealed that individuals with lower socioeconomic status (SES) exhibit poorer responsiveness to available interventions and a higher prevalence of smoking-related comorbidities compared to their higher SES counterparts. Furthermore, gender differences are noteworthy, with women, particularly those of lower SES, facing greater challenges in quitting due to factors such as smoking dependency, limited access to interventions, stress-coping mechanisms, time constraints, and concerns about the financial implications of smoking cessation [18].

These unique challenges emphasize the need for modified interventions tailored to each socioeconomic group, healthcare institution, and geographical area. The global burden from smoking calls for the urgent implementation of these interventions.

Tailoring interventions for diverse populations

It is crucial to understand that a one-size-fits-all approach does not work when it comes to smoking cessation. To increase the effectiveness of smoking cessation interventions, we need to consider the the below-mentioned factors.

Gender Differences

Understanding the unique communication styles and preferences between men and women is essential in smoking cessation.

According to a study by Dieleman et al., women may find more benefit from emotionally supportive counseling, while men may respond better to goal-oriented and competitive strategies. By acknowledging and catering to these diverse preferences, personalized interventions can effectively help individuals overcome challenges and succeed in their journey to quit smoking [19].

Smith et al. conducted a meta-analysis emphasizing the importance of considering gender when offering NRT. The study found that women using varenicline had a 51% higher chance of quitting smoking compared to those using a transdermal nicotine patch, while no significant differences were observed among men. This suggests that gender-specific responses to smoking cessation medications may exist, possibly influenced by factors such as drug metabolism and smoking-related cues. Taking these findings into account can assist in tailoring treatment for individuals [20].

Cultural Considerations

Cultural diversity plays a vital role in smoking behavior, and it is crucial for interventions to be sensitive to these differences. Castro et al. elucidated that when immigrants adapt to a new culture, it can influence their smoking patterns. The study discovered that the interaction between American and Mexican cultural identity significantly impacted smoking abstinence, with greater American cultural identity associated with abstinence-only among those with high Mexican cultural identity. These compelling findings underline the importance of embracing multiple cultures in the context of smoking cessation interventions. By tailoring our approaches and support to the unique needs and perspectives of diverse communities, we can foster a more inclusive and effective environment for individuals striving to quit smoking [21].

Age-Specific Strategies

Different age groups face unique challenges and motivations when it comes to quitting smoking. Kim et al. using data from the Korea National Health and Nutrition Examination Survey reported that factors associated with successful smoking cessation varied among different age groups. Their research, spanning from 2007 to 2018, showed varying success rates for quitting across age categories: 31.5% for young adults (aged 19-39 years), 54.7% for middle-aged individuals (aged 40-64 years), and 78.6% for older adults (aged 65 years and above). The determinants of successful quitting were age-dependent. Marriage was associated with successful cessation in young adults and middle-aged individuals, while a lifelong history of smoking hindered quitting efforts. Willpower and specific medical conditions were linked to successful cessation in middle-aged and older adults. Conversely, unhealthy behaviors such as meal skipping were negatively correlated with quitting among young adults. These findings emphasize the importance of taking age-specific factors into account when devising smoking cessation policies and programs [22].

Future directions

Smoking continues to pose a substantial global public health challenge, contributing significantly to both morbidity and mortality rates. In the past, smoking cessation efforts relied on generic, one-size-fits-all strategies, yielding mixed results among individuals. Acknowledging these limitations, there is now a growing push toward personalized approaches to smoking cessation. This shift has sparked exploration into cutting-edge technologies aimed at tackling the intricate nature of smoking addiction and enhancing tailored support. Additionally, there are emerging trends in tobacco control policies that are shaping the landscape of smoking cessation efforts.

In the future of smoking cessation, innovative technologies will play a pivotal role in streamlining the process. AI-driven interventions will meticulously analyze individuals’ smoking patterns, triggers, and cravings. They will provide real-time, adaptive support through chatbots and virtual coaches, delivering personalized guidance and motivational messages to bolster quitting efforts [23].

Furthermore, the integration of data from mobile phones and smartwatches will create an ideal platform for offering immediate feedback on stress levels and cravings. This data-driven approach will empower personalized interventions, further enhancing the effectiveness of smoking cessation strategies [23].

Innovative techniques for modulating brain activity without surgical interventions, such as repetitive transcranial magnetic stimulation (rTMS) or transcranial direct current stimulation (tDCS), have shown promise in reducing nicotine cravings and cigarette consumption. In a study by Coles et al., participants experienced a notable decrease in nicotine cravings and an overall reduction in cigarette smoking following the application of rTMS and tDCS. These methods work by targeting addiction-related brain areas, making the journey to quit smoking more attainable. Looking ahead, there is potential for these tools to become more readily available, offering valuable assistance in smoking cessation efforts [24].

Gaining a comprehensive grasp of the interplay between genetics and metabolism is crucial in the context of smoking cessation. As elucidated in the study conducted by Chen et al., individuals possessing a high-risk genetic haplotype demonstrated a threefold greater likelihood of responding positively to pharmacologic cessation treatments when juxtaposed with those harboring a low-risk haplotype. This revelation outlines the pivotal role of genetic knowledge in the tailoring of precise and personalized smoking cessation interventions. Such interventions take into account an individual’s susceptibility to smoking initiation, inherent predisposition to nicotine addiction, propensity for achieving successful cessation, and unique response to treatment modalities. The amalgamation of these insights not only provides a deeper understanding of the intricate dynamics at play but also augments the potential for more widespread utilization of personalized approaches in the future [25].

In light of future trends in smoking cessation and the incorporation of technologies such as artificial intelligence and brain stimulation, it becomes evident that innovative and forward-thinking policies are essential drivers of change in smoking cessation programs. Drawing inspiration from the SimSmoke model, this review explores visionary approaches poised to revolutionize the landscape of smoking cessation efforts. These futuristic policies aim to address the complexities of smoking addiction and offer tailored solutions for individuals striving to quit.

According to Levy et al., the SimSmoke model predicts that a $1.00 tax increase applied to an initial price of $2.00 would yield a 13% reduction in the prevalence of cigarette smoking among adults after five years (short-term) and a 30% reduction after 40 years [26]. These policies not only motivate quitting and reduce consumption but also generate revenue to support tobacco control, including cessation services. As policymakers contemplate raising prices of tobacco product, they should ensure securing adequate funding and availability of cessation services to meet the growing demand [27].

While smoke-free laws primarily aim to protect non-smokers from secondhand smoke, they have also been linked to decreased smoking prevalence, reduced cigarette consumption, and increased cessation. The SimSmoke model predicts a 10% short-term and 13% long-term reduction in smoking prevalence [28] with comprehensive smoke-free laws, potentially leading to 14.7% of current smokers quitting if all U.S. indoor workplaces became smoke-free [27].

Research indicates a dose-response connection between greater exposure to emotionally compelling mass media campaigns depicting the health risks of smoking and higher quit rates and quit line calls [27]. According to the SimSmoke model, large-scale mass media campaigns can lead to a 6% short-term and 10% long-term reduction in smoking prevalence [28].

Evidence indicates that systematically lowering nicotine levels in cigarettes could help prevent the development of nicotine addiction. This approach may increase the chances of addicted adult smokers quitting, as reducing nicotine content would make cigarettes less addictive and less satisfying, thus reducing nicotine dependence and promoting tobacco abstinence [27]. Although research is still ongoing on the use of e-cigarettes in smoking cessation, few studies have shown positive responses [29]. However, due to some inconsistencies in available data, further research is required regarding the long-term implications of exposure to e-cigarettes.

Smoking cessation remains a complex and challenging endeavor, but with innovative approaches, technologies, and collaborative efforts from policymakers, advancements can be made to further reduce smoking rates, support cessation efforts, and reduce the global burden of smoking-related diseases.

## Conclusions

Smoking is a major global health concern, claiming countless lives annually. Notably, a combination of pharmacological and behavioral therapies has emerged as the most successful approach to quitting smoking. However, the effectiveness of these interventions is influenced by a multitude of factors, including genetic predisposition, individual characteristics, and SES. Acknowledging these factors is important in tailoring interventions to diverse demographics. The implementation of evidence-based interventions, targeted strategies, and modern technology holds the promise of further reducing smoking rates, bolstering cessation efforts, and ultimately alleviating the global burden of tobacco-related diseases. We recommend healthcare workers, governments, and other key stakeholders maintain their collaborative efforts and adopt various strategies for smoking cessation to enhance public health outcomes.
